# Discontinuation of Long-acting Injectable Cabotegravir–Rilpivirine in a Large Clinic Cohort

**DOI:** 10.1093/ofid/ofaf600

**Published:** 2025-09-26

**Authors:** Katerina A Christopoulos, Xavier Erguera, Janet Grochowski, Matthew Hickey, Elizabeth Imbert, Samantha Dilworth, Ayesha Appa, Chesa Cox, Mary Shiels, Jon Oskarsson, Monica Gandhi

**Affiliations:** Division of HIV, ID and Global Medicine, University of California San Francisco, San Francisco, California, USA; Division of HIV, ID and Global Medicine, University of California San Francisco, San Francisco, California, USA; Division of HIV, ID and Global Medicine, University of California San Francisco, San Francisco, California, USA; Division of HIV, ID and Global Medicine, University of California San Francisco, San Francisco, California, USA; Division of HIV, ID and Global Medicine, University of California San Francisco, San Francisco, California, USA; Division of Prevention Science, University of California San Francisco, San Francisco, California, USA; Division of HIV, ID and Global Medicine, University of California San Francisco, San Francisco, California, USA; Division of HIV, ID and Global Medicine, University of California San Francisco, San Francisco, California, USA; Division of HIV, ID and Global Medicine, University of California San Francisco, San Francisco, California, USA; Division of HIV, ID and Global Medicine, University of California San Francisco, San Francisco, California, USA; Division of HIV, ID and Global Medicine, University of California San Francisco, San Francisco, California, USA

**Keywords:** discontinuation, HIV/AIDS, injectable cabotegravir and rilpivirine, long-acting antiretroviral therapy, persistence

## Abstract

Little is known about long-acting injectable cabotegravir-rilpivirine discontinuation outside clinical trials. In a large clinic cohort, 1 in 6 people with HIV discontinued long-acting injectable cabotegravir-rilpivirine over a median of ∼18 months. Injection-related pain was common. Virologic failure and efficacy concerns drove discontinuation for people with HIV with adherence challenges. Most had viral suppression on alternate antiretrovirals post-discontinuation.

## Teaser

In a large U.S. based clinic cohort of people with HIV (PWH) using long-acting cabotegravir-rilpivirine, 1 out of 6 discontinued over a median follow-up of eighteen months. Viral suppression on alternate antiretroviral therapy after discontinuation was high, even in the small number of cases with treatment-emergent resistance mutations.

Long-acting injectable cabotegravir–rilpivirine (CAB/RPV-LA) is the first complete long-acting regimen for the treatment of HIV-1. People with HIV (PWH) and their providers have been enthusiastic about CAB/RPV-LA [1, 2], which is particularly beneficial for PWH who face challenges to daily oral antiretroviral therapy (ART) adherence [3]. However, focused inquiry into persistence on CAB/RPV-LA and reasons for treatment discontinuation is lacking. Industry-sponsored trials of CAB/RPV-LA in treatment-experienced PWH report discontinuation rates after 1–2 years of ∼10% [4, 5], with injection site reactions and other adverse events being the most common reasons for discontinuation. In the LATITUDE study, which randomized PWH with adherence challenges to CAB/RPV-LA vs. continued oral ART, 28/146 (19%) discontinued CAB/RPV-LA, including 5 (3%) with virologic failure [6]. To determine whether CAB/RPV-LA discontinuation in clinical practice is similar to trial findings, we sought to investigate frequency of and reasons for discontinuation in a large clinic-based cohort. In addition, given that the long pharmacokinetic tail of CAB/RPV-LA raises concern for evolution of HIV viral resistance if alternate ART is not initiated promptly, we also assessed postdiscontinuation viral suppression and development of resistance mutations.

## METHODS

We conducted a retrospective study of PWH who discontinued CAB/RPV-LA at the Ward 86 clinic in San Francisco. Ward 86 serves low-income publicly insured PWH, many of whom face adherence challenges such as substance use, mental illness, and homelessness. The CAB/RPV-LA program was created in June 2021 and from its inception allowed PWH with viremia due to adherence challenges to initiate CAB/RPV-LA [3]. For this analysis, we included all PWH who received ≥1 dose of CAB/RPV-LA and subsequently discontinued, censoring data on 26 January 2025. We defined discontinuation as intended cessation of CAB/RPV-LA without documented plan to resume or loss to follow-up per clinical notes. Deaths occurring while PWH were using CAB/RPV-LA were not considered discontinuations.

Using pharmacy team logs to identify PWH who discontinued CAB/RPV-LA, 2 members of the study team (K.A.C., X.A.E.) abstracted reasons for discontinuation from the electronic medical record (EMR) and summarized these data using content analysis. Demographics and clinical variables were also abstracted from the EMR. Viral suppression (VS) after discontinuation was defined as viral load (VL) < 200 copies/mL and ascertained for those with at least 90 days of follow-up after their last injection and those with ≥1 year of follow-up; reasons for missing VL measurements were abstracted from the EMR. Descriptive statistics summarized demographic and clinical characteristics, stratified by VS status at first CAB/RPV-LA injection, with VS at initiation defined as <50 copies/mL and viremia as ≥50 copies/mL per the criteria used in clinical trials [4, 5]. We also explored bivariate associations between baseline characteristics and discontinuation, including the potential for moderation by male sex, with results presented as odds ratios with 95% confidence intervals and *P* values.

The University of California San Francisco Institutional Review Board approved this study.

## RESULTS

Between June 2021 and January 2025, 438 PWH received ≥1 dose of CAB/RPV-LA with median time on injections of 18.2 months (interquartile range 8.3, 26.5 months). In this cohort, 69 (16%) discontinued, of whom 47 (68%) initiated CAB/RPV-LA with VS and 22 (32%) with viremia. Discontinuation rates were similar for those starting with VS (47/289, 16%) and with viremia (22/149, 15%), chi-squared *P* = .68. The median age of those who discontinued was 44 years (interquartile range 34–56 years), median CD4 cell count was 478 cells/mm [3], 90% were male, 33% Latino, 16% Black, 46% experiencing unstable housing or homelessness at CAB/RPV-LA referral, and 36% were using stimulants (see Supplementary Table 1 for characteristics of PWH who discontinued vs. persisted with CAB/RPV-LA).

At discontinuation, the median number of injections received was 6 (min 1, max 32) with 30% on every 8-week dosing. Seven individuals (10%) discontinued after 1 injection and 21 (30%) after ≥10 injections. Overall, reasons for discontinuation (Table 1) were categorized into: (1) treatment-related considerations (e.g., injection-related pain, other concern/side effect) for 43/69 (62%); (2) efficacy considerations (i.e., virologic failure or late injections leading to provider concern about continued efficacy) for 16/69 (23%); (3) logistical considerations beyond the patient's control (e.g., incarceration, relocation) for 7/69 (10%); and (4) loss to follow-up for 3/69 (5%). For those initiating with VS, the most common reason for discontinuation was pain, with or without another side effect or concern (Table 1). One patient initiating CAB/RPV-LA with VS developed the K101E mutation with repeated late injections but resuppressed on oral ART (Supplementary Table 2). For those initiating with viremia, episodes of lateness raised provider concern for efficacy and virologic failure drove discontinuation. Virologic failure with nonnucleoside reverse transcriptase inhibitor and/or integrase strand transfer inhibitor resistance-associated mutations (RAMs) led to discontinuation for 6 PWH (2 with on-time injections; 4 in the setting of late injections); all started alternate ART and at database closure 3 had resuppressed, 2 were awaiting laboratory results, and 1 was lost to follow-up (Supplementary Table 2). Another 6 PWH were lost to follow-up or declined ART after discontinuing CAB/RPV-LA and subsequently developed nonnucleoside reverse transcriptase inhibitor and/or integrase strand transfer inhibitor RAM. All eventually received alternate ART and resuppressed, except for the individual who declined ART.

**Table 1.** Reasons for Discontinuation and Subsequent HIV Viral Load (VL) Outcomes, Stratified by HIV VL <50 copies/mL vs. ≥50 copies/mL at Long-acting Cabotegravir–Rilpivirine Initiation

	Overall (n = 69)	HIV VL <50 copies/mL (n = 47)	HIV VL ≥50 copies/mL (n = 22)
Reason for discontinuation			
Treatment-related consideration			
Injection site pain	14 (20.3)	10 (21.3)	4 (18.2)
Injection site pain with other side effect/concern^a^	11 (15.9)	9 (19.1)	2 (9.1)
Other side effect/concern^a^	14 (20.3)	11 (23.4)	3 (13.6)
Need to come to clinic for injections	2 (2.9)	2 (4.3)	
Allergic reaction	1 (1.4)	1 (2.1)	
Declined antiretroviral therapy but remained in care	1 (1.4)		1 (4.5)
Efficacy consideration			
Virologic failure	7 (10.1)	1 (2.1)	6 (27.3)
≥1 episode of lateness leading to provider concern for continued efficacy of regimen	8 (11.6)	2 (4.3)	6 (27.3)
Provider discontinuation for HIV RNA blip	1 (1.4)	1 (2.1)	
Logistical consideration			
Residential treatment for mental health/substance use or incarceration	5 (7.2)	5 (10.6)	
Relocation	2 (2.8)	2 (4.3)	
Loss to follow-up	3 (4.3)	3 (6.4)	
HIV VL outcomes after discontinuation			
Has ≥90 d follow-up until database closure	65/69 (94.2)	45/47 (95.7)	20/22 (90.9)
Has VL measure after discontinuation^b^	51/65 (78.5)	34/45 (75.6)	17/20 (85.0)
Last VL <200 copies/mL	48/51 (94.1)	34/34 (100)	14/17 (82.4)
Has >365 d since last injection	44/69 (63.8)	29/47 (61.7)	15/22 (68.2)
Has VL measure >365 d after last injection^c^	26/44 (59.1)	13/29 (44.8)	13/15 (86.7)
Last VL >365 d from last injection has VS	24/26 (92/3	13/13 (100)	11/13 (84.6)

^a^Other side effect/concern (not mutually exclusive): flu-like symptoms (7), weight gain (2), fatigue (2), patient concern about efficacy (3), mistrust/misunderstanding (2), muscle spasms (1) injection site abscesses (1), bloating (1), sleep/appetite concern (1), patient desire for control of HIV treatment (1), feeling “stuck in a jar” (1), wanted to “take a break” (1), feeling like “too much medicine” in body (1), discomfort with subcutaneous lenacapavir injections given for intensification of treatment regimen.

^b^Reason for absence of follow-up VL in those with >90 d since last injection: loss to follow-up (7), relocation (3), patient deferral of venipuncture (3), incarceration (1).

^c^Reason for absence of VL measurement >365 d since last injection among those with adequate follow up time: no VL measurement >90 d after last injection, included in group above (7), chart notes indicative of engagement and/or adherence to ART (9), subsequent loss to follow-up after initial VS (2).

For the overall cohort, of those with ≥90 days follow-up time after the last injection, 51/65 had any VL measured with 48/51 (94%) achieving VS (Table 1). Forty-four of these individuals had ≥1 year of follow-up. Although only 26 had a VL measured more than 1 year after CAB/RPV-LA discontinuation, 24/26 (92%) had VS. Of the 18 individuals without VL measurement, 7 did not have any VL measurement after discontinuation due to loss to follow up (n = 4), relocation (n = 2), and incarceration (n = 1). Of the remaining 11 cases, all had evidence of VS after discontinuation with no documented VLs >200 copies/mL; chart review indicated that ≥1 year after discontinuation, 9/11 were engaging in care and/or adhering to ART and 2/11 were lost to follow-up. Thus, a reasonable lower bound for an estimate of longer-term VS after discontinuation is 33/44 (75%). This estimate would be higher if some of those with unobserved outcomes due to relocation, incarceration, or lost to follow-up are considered to have VS.

Finally, in bivariate analyses, we found that the odds of discontinuation were twice as high in men (odds ratio, 2.17; 95% confidence interval [CI], 1.04–5.22; *P* < .01) and in those using stimulants at the time of CAB/RPV-LA referral (odds ratio, 2.03; 95% CI, 1.16–3.48; *P* < .01) with no other variables having *P* < .05. In a multivariable model including sex and stimulant use at CAB/RPV-LA referral, only stimulant use remained significant (adjusted odds ratio, 1.97; 95% CI, 1.12–3.39; *P* < .02) with no moderation by sex (Supplementary Table 3).

## DISCUSSION

In a large cohort of CAB/RPV-LA users with median time on injections of approximately 18 months, discontinuation occurred in 16%, with similar proportions in those who initiated with VS and those who initiated with viremia. These findings are slightly higher than the rate observed in clinical trials (∼10% after 1–2 years) but comparable to the rate of 13%–14% found in other academic Ryan White–funded clinics in Atlanta [7] and Chicago [8] and a large prospective U.S. multicenter observational cohort [9]. In our dataset, treatment-related considerations (e.g., injection-related pain) accounted for nearly two-thirds of discontinuation.

Indeed, among people initiating CAB/RPV-LA with VS, the most common reason was injection site pain, with or without another side effect or concern. Our qualitative research with PWH who have discontinued CAB/RPV-LA has found that for those who had VS on oral ART without adherence challenges the benefits of CAB/RPV-LA may not outweigh the burden of “gearing up for” and experiencing pain, both during and after injections [10]. In addition, some PWH in this study reported other treatment-related side effects, including flu-like symptoms and fatigue. Mistrust resulting from a misunderstanding of CAB/RPV-LA treatment appeared to be a factor for only 2 PWH, and only 2 PWH cited the need to come to clinic as the reason for discontinuation.

In PWH initiating CAB/RPV-LA with viremia due to adherence challenges, about half of discontinuations were due to virologic failure or provider concern for continued efficacy with repeated late injections. These findings align with those from a study of 62 PWH with adherence challenges initiating CAB/RPV-LA at the University of California San Diego, in which discontinuation by 48 weeks occurred in ∼18%, with about half of discontinuations from virologic failure [11]. In our cohort, discontinuation from a treatment-related consideration was lower (45%) for those initiating CAB/RPV-LA with viremia compared to those with VS (62%). Indeed, PWH with adherence challenges may be more likely to persist with CAB/RPV-LA because of decreased anxiety related to pill-taking, increased self-worth upon achieving VS, and better health status after VS [10]. It is worth noting that although 6 PWH developed RAMs after discontinuation in our study, 5 of 6 who accepted alternate ART resuppressed.

Although 10% of our study population discontinued CAB/RPV-LA after the first injection, nearly one-third discontinued after ≥10 injections. As PWH accrue more time on injections, closer examination of the reasons for discontinuation after years of treatment will be necessary to optimize transitions to alternative treatment. Finally, our data showed that stimulant use at CAB/RPV-LA referral was associated with discontinuation; however, we do not have data on stimulant use more proximal to the time of discontinuation or other time-updated sociostructural variables that could affect persistence, such as housing status or depressive symptoms, thus these results should be interpreted with caution.

Limitations of this study include a single site, which may limit generalizability. Indeed, important reasons for discontinuation that we did not find but that have been noted by others are cost and access [7, 9]. Because Medicaid and the AIDS Drug Assistance Program in California generally provide access to CAB/RPV-LA without need for prior authorizations or copays, PWH at Ward 86 have not faced cost/access barriers, which can be formidable in other parts of the country or for those with commercial insurance [12]. In addition, Ward 86 has resources (e.g., low-barrier drop-in primary care to support viral suppression after discontinuation) that may not be available in other clinics. Reasons for discontinuation were obtained via chart review, which reflects only provider understanding of the discontinuation. If queried directly, it is possible that PWH may report different reasons; as such, we are undertaking additional qualitative research with PWH. Finally, ∼40% of PWH with adequate follow up time did not have a viral load measurement >1 year after discontinuation, which reflects loss to follow-up, relocation, and less frequent laboratory monitoring in those who resume oral ART successfully; however, chart notes indicate that most PWH were engaging with care and adhering to ART. More complete laboratory measurement would allow a more robust characterization of clinical outcomes after CAB/RPV-LA discontinuation.

In summary, we found that over a median of 18 months, nearly 1 of 6 people initiated on CAB/RPV-LA in a real-world cohort discontinued it. Injection-related pain drove discontinuation for PWH who initiated CAB/RPV-LA with VS, while virologic failure and episodes of lateness raising provider concern for continued efficacy accounted for most discontinuation in those initiating with viremia. However, reassuringly, most PWH, including those with RAMs, achieved VS on alternate ART.

## Supplementary Material

ofaf600_Supplementary_Data

## Supplementary Data

Supplementary materials are available at *Open Forum Infectious Diseases* online. Consisting of data provided by the authors to benefit the reader, the posted materials are not copyedited and are the sole responsibility of the authors, so questions or comments should be addressed to the corresponding author.
