# Supplementary material for: Discontinuation of Long-acting Injectable Cabotegravir–Rilpivirine in a Large Clinic Cohort
Source: Open Forum Infect Dis. 2025 Sep 26;12(10):ofaf600. doi: 10.1093/ofid/ofaf600 (PMC12534727; doi:10.1093/ofid/ofaf600)
Supplement: ofaf600_Supplementary_Data [file ofaf600_supplementary_data.zip › Supplemental Table 1.docx]

Supplemental Table 1. Characteristics of People with HIV Who Received ≥1 Injection of Long-Acting Cabotegravir/Rilpivirine (CAB/RPV-LA) N=438

|  | Discontinued CAB/RPV-LA (N=69) N (%) | Remained on CAB/RPV-LA (N=369) N (%) |
| --- | --- | --- |
| Age, median (IQR) | 44 (34 – 56) | 46 (37 – 56) |
| Categorized Age |  |  |
| 18-29 | 1 (1.5) | 18 (4.9) |
| 30-40 | 43 (62.3) | 193 (52.3) |
| 50+ | 25 (36.2) | 158 (42.8) |
| Race/Ethnicity |  |  |
| White | 22 (31.9) | 123 (33.3) |
| Black | 11 (15.9) | 74 (20.1) |
| Latino/a/x | 23 (33.3) | 113 (30.6) |
| Other | 13 (18.8) | 59 (16.0) |
| Sex/Gender |  |  |
| Female | 6 (8.7) | 43 (11.7) |
| Male | 62 (89.7) | 293 (79.4) |
| Gender minority | 1 (1.5) | 33 (8.9) |
| Housing Status at CAB/RPV Referral |  |  |
| Stable | 37 (53.6) | 229 (62.1) |
| Unstable | 24 (34.8) | 113 (30.6) |
| Homeless | 8 (11.6) | 27 (7.3) |
| Stimulant Use at CAB/RPV Referral | 25 (36.2) | 81 (22) |
| Viral Suppression at CAB/RPV Initiation | 47 (68.1) | 242 (65.6) |
