# Supplementary material for: Discontinuation of Long-acting Injectable Cabotegravir–Rilpivirine in a Large Clinic Cohort
Source: Open Forum Infect Dis. 2025 Sep 26;12(10):ofaf600. doi: 10.1093/ofid/ofaf600 (PMC12534727; doi:10.1093/ofid/ofaf600)
Supplement: ofaf600_Supplementary_Data [file ofaf600_supplementary_data.zip › Supplemental Table 2.docx]

Supplemental Table 2. Adverse CAB/RPV-LA Virologic Outcomes

| **Initiated with Viral Suppression (VS) – Developed Resistance Mutations While Receiving CAB/RPV-LA With Late Injections** | | | | | | |
| --- | --- | --- | --- | --- | --- | --- |
|  | **Baseline CD4/VL** | **Baseline RAMs** | **# of Injections and Lateness** | **New RAMs** | **Alternate ART** | **VS Post Discontinuation** |
| Patient 1 | CD4 395; VL not detected | n/a | Initiated q4wk dosing, late at 4^th^, 6^th^ injections, reloaded when 50d late for 8^th^ injection🡪 q8week dosing, 11d late to 11th injection, 42d late to 14^th^ and genotype done with VL=62 | K101E | TAF/FTC/DRV/c | Yes |
| **Initiated with Viremia – Developed Resistance Mutations While Receiving CAB/RPV-LA** | | | | | | |
|  | **Baseline CD4/VL** | **Baseline RAMs** | **# of Injections and Lateness** | **New RAMs** | **Alternate ART** | **VS Post Discontinuation** |
| Patient 2 (failure with on-time injections) | CD4 15; VL 137,000 | T97A (minor INSTI) | Initiated q4wk dosing, VL 4,400 at wk 4 | E138 K (NNRTI) and R263K (INSTI) | Initially disengaged from care but returned and started BIC/TAF/FTC + LEN | Yes |
| Patient 3 (failure with on-time injections) | CD4 71; VL 215,000 | V179I and N348I (minor NNRTI) | Initiated q4 week dosing, VL 29,000 at wk 4 | L100I and Y181I mutations (NNRTI) | Initially disengaged from care but later returned and started CAB + LEN | Yes |
| Patient 4 (failure with late injections) | CD4 306; VL 363,800 | n/a | Had 9 on-time q4wk injections then 🡪 q8wk; 10d late to 10th injection, VL 29,000; Repeat VL 6d later 79. Thought to be a lab error/patient wanted to continue LA-CAB/RPV. Subsequently received 3 on-time q8wk injections but had VL 4,500 at the 13th injection (wk 68) | K101E and Q148R | CAB/RPV + LEN 🡪 TAF/FTC/BIC | Has not yet had VL measurement |
| Patient 5 (failure with on time injections) | CD4 731; VL 700 | K103N, T369V, I178I/M | Received 8 on-time q4wk injections via home nursing. At 5^th^ injection VL 80; At 8^th^ injection noted to have VL 1,300 | NNRTI: K101K/E, K103N, I178M, Y181Y/C, V189V/I, T369V INSTI E138E/K, Q148K. | TAF/FTC/DRV/c. | Yes |
| Patient 6 (failure with late injections) | CD4 83; VL 309 | M184V | Initiated with q4wk injections. Was 12d late for 4th injection and had VL 66; genotype failed. VS at 5^th^ injection 🡪 q8wk dosing. At 7^th^ injection VL 137,000; at 8^th^ injection VL 256,000 | M230L | Care team confirmed patient picked up TAF/FTC/DRV/c from pharmacy | Lost to follow up |
| Patient 7 (failure with late injections) | CD4 150; VL 540,000 | V90I | Initiated q4wk then VS, had blips of 88 and 33 🡪 q8wk at 5^th^ injection. VS at 6^th^injection. 9d late to 7^th^ injection, 13d late to 9^th^ injection and VL 8,600. | RT I178L, F127V, M230L, K101Q, INSTI: E138E/D/K/N, G140G/S, S147S/G, Q148K. | TAF/FTC/DRV/c + LEN. | Repeat VL ordered but not yet completed. |
| **Initiated with Viremia and Developed Resistance After CAB/RPV-LA Discontinuation** | | | | | | |
|  | **Baseline CD4/VL** | **Baseline RAMs** | **# of Injections and Lateness** | **New RAMs** | **Alternate ART** | **VS Post Discontinuation** |
| Patient 8 | CD4 20; VL 67,000 | None | Received 10 on-time injections; VL <200 copies/mL wk 4 and <50 wk 24; self-discontinued due to severe depression | NNRTI: K101E, E138 K, Y181F/I/N, M230L | None | No |
| Patient 9 | CD4 27; VL 801,000 | V179I | Received 5 on-time q4wk injections; was 15d late to 6th and received reloading. Presented on time for 7^th^ injection but left without being seen, then discontinued all ART due to belief they were cured of HIV; declined all care engagement until 1 y later; VL 226,000 | RT E138A/K/T and Y181C | CAB + LEN | Yes |
| Patient 10 | CD4 10; VL 34,000 | M184V | Received 7 on-time q4 wk injections; 14d late to 8^th^ injection and when came to clinic 🡪 TAF/FTC/DRV/c due to c/f SSTI. VS observed 3 months later, then patient discontinued oral ART and was LTFU, returned and started TAF/FTC/BIC | Genotype 14 months after last injection K101E, V179I, Y181Y/C | TAF/FTC/DRV/c 🡪 TAF/FTC/BIC | Yes |
| Patient 11 | CD4 50; VL 114,000 | None | Had 17 q4 wk injections with 6 late, one requiring a reloading. VL 382 and switched to TAF/FTC/DRV/r due to concern for resistance. Patient disengaged from care and presented one year later with VL 12,000. | K101E. | TAF/FTC/DRV/r 🡪 CAB + LEN with home nursing | Yes |
| Patient 12 | CD4 56; VL 1,700 | n/a  (History of TDF/FTC/RPV use) | Had 17 on-time q4wk injections, then 62d late, reloaded; on-time at wk4; then reloaded at 67d late. At last injection VL 99 and genotype with K101E, then disengaged from care | K101E; then disengaged from care and returned 5 months later with E138K (NNRTI) | TAF/FTC/DRV/c | Yes |
| Patient 13 | CD4 20; VL 18,600 | n/a | Had 2 on-time injections and then disengaged. Returned to clinic two years later with VL 5,000 | V106VI, Y181Y/C, H221Y, I178I/M | TAF/FTC/DRV/c | Yes |
| **Initiated With Viremia – Resistance Not Confirmed** | | | | | | |
| Patient 14 | CD4 25; VL 140 | None | Received 3 on-time q4wk injections, VL at 3^rd^ injection came back at 16,000. | VS before genotype obtained | TAF/FTC/DRV/c | Yes |
| *Cases shaded in gray described in Hickey et. al., CID 2024, Gandhi et. al., Annals Int Med, 2023, and Christopoulos et. al., CID 2022. | | | | | | |
