# Supplementary material for: Discontinuation of Long-acting Injectable Cabotegravir–Rilpivirine in a Large Clinic Cohort
Source: Open Forum Infect Dis. 2025 Sep 26;12(10):ofaf600. doi: 10.1093/ofid/ofaf600 (PMC12534727; doi:10.1093/ofid/ofaf600)
Supplement: ofaf600_Supplementary_Data [file ofaf600_supplementary_data.zip › Supplemental Table 3.docx]

Supplemental Table 3. Association of Baseline Characteristics with CAB/RPV-LA Discontinuation

|  | Unadjusted | | Adjusted | |
| --- | --- | --- | --- | --- |
|  | Odds Ratios  (95% CI) | p-value | Odds Ratios (95% CI) | p-value |
| Age *(increments of 5 years)* | 0.95 (0.86, 1.06) | 0.38 | -- | -- |
| Race/Ethnicity *χ^2^ (df)* | 0.96 (3) | 0.82 | -- | -- |
| White | -- | -- | -- | -- |
| Black | 0.85 (0.38, 1.79) | 0.67 | -- | -- |
| Latino/a/x | 1.14 (0.60, 2.15) | 0.69 | -- | -- |
| Other | 1.25 (0.58, 2.59) | 0.56 | -- | -- |
| **Male Sex** | **2.17 (1.04, 5.22)** | **0.04** | 2.09 (0.99, 5.04) | 0.053 |
| Housing at CAB/RPV-LA Referral *χ^2^ (df)* | 2.48 (2) | 0.29 | -- | -- |
| Stable | -- | -- | -- | -- |
| Unstable | 1.32 (0.75, 2.29) | 0.33 | -- | -- |
| Homeless | 1.89 (0.77, 4.26) | 0.15 | -- | -- |
| **Stimulant Use at CAB/RPV-LA Referral** | **2.03 (1.16, 3.48)** | **0.01** | **1.97 (1.13, 3.39)** | **0.02** |
| Viral Suppression at CAB/RPV-LA Initiation | 0.90 (0.51, 1.54) | 0.71 | -- | -- |
